# Evolution of gene order in mtDNA of Baikal endemic amphipods and its possible mechanisms

**DOI:** 10.18699/vjgb-26-15

**Published:** 2026-03

**Authors:** E.A. Sirotinina, D.Yu. Sherbakov, E.V. Romanova

**Affiliations:** Limnological Institute of the Siberian Branch of the Russian Academy of Sciences, Irkutsk, Russia; Limnological Institute of the Siberian Branch of the Russian Academy of Sciences, Irkutsk, Russia; Limnological Institute of the Siberian Branch of the Russian Academy of Sciences, Irkutsk, Russia

**Keywords:** amphipods, Lake Baikal, mitochondrial genome, gene rearrangement, амфиподы, озеро Байкал, митохондриальный геном, перестройки генов

## Abstract

Significant gene order diversity of mitochondrial (mt) genomes of invertebrates is peculiar to subphylum Crustacea, and to order Amphipoda in particular. Amphipods from Lake Baikal are also known as a group with unique gene orders in their mt genomes. To estimate the diversity of protein-coding gene orders (GOs) in amphipods, a comparative analysis of gene rearrangements in the mt genomes of Baikal and non-Baikal species was performed. In some cases, gene rearrangement data and the history of gene relocation in different taxonomic groups can also supplement the results of phylogenetic inferences. Among the thirteen mt genomes of Baikal species sequenced in previous studies, four gene order patterns were identified, and fourteen gene order patterns for 114 mt genomes of non-Baikal species were observed. The type and number of rearrangement steps (from 1 to 3) required to transition from one order to another and the number of mt genes rearranged in each GO (from 1 to 5) were also defined. Baikalian amphipods belong to two lineages (I and II) according to molecular data which reveal their origin from two independent introductions of ancestral species into the lake. All cases of mt gene order rearrangements have been detected in species from the first lineage, whereas the mt gene order in the second lineage is conserved in all species studied and corresponds to the Pancrustacean pattern (PanGO). PanGO has been determined as the ancestral gene order for both Baikalian amphipod lineages. The possible mechanisms of mt gene order rearrangements such as a complete or partial duplication of mt genome and subsequent random deletions are discussed in our study. It is supposed that increased mutation rate, weakening of stabilizing selection and other specific factors may influence the probability of emergence and fixation of different GOs in mt genomes of Baikalian amphipods

## Abstract (ru)

Значительное разнообразие в порядке генов митохондриальных (мт) геномов беспозвоночных отмечается в подтипе Crustacea, и, в частности, в отряде Amphipoda. Амфиподы озера Байкал также известны как группа с уникальными порядками генов в мт геномах. Чтобы оценить разнообразие порядков белок-кодирующих генов (ПГ) амфипод, был проведен сравнительный анализ генных перестроек в мт геномах байкальских и небайкальских видов. В некоторых случаях данные о перестройках генов и истории их перемещений у различных таксономических групп также могут информативно дополнять данные филогенетического анализа. Для 13 ранее полученных нуклеотидных последовательностей мт геномов байкальских видов мы определили 4 варианта ПГ, для 114 мт геномов небайкальских видов – 14 вариантов ПГ. Были также рассчитаны типы и число шагов перестроек (от 1 до 3), требующихся для перехода от одного порядка генов к другому, и число мт генов, подвергшихся перестройкам в каждом ПГ (от 1 до 5). Байкальские амфиподы принадлежат к двум линиям (I и II) в соответствии с молекулярными данными, указывающими на их происхождение от двух независимых вселений предковых видов в озеро. Все случаи перестроек порядка мт генов обнаружены у видов из линии I, тогда как порядок мт генов в линии II консервативен у всех изученных видов и соответствует модели Pancrustacean pattern (ПанПГ). ПанПГ был определен как предковый порядок генов для обеих линий байкальских амфипод. В исследовании обсуждаются возможные механизмы перестроек порядка мт генов, такие, как полная или частичная дупликация мт генома и последующие случайные делеции. Высказывается предположение, что повышенная скорость мутаций, ослабление стабилизирующего отбора и другие особые факторы могут влиять на вероятность появления и фиксации различных ПГ в мт геномах байкальских амфипод.

## Introduction

The diversity of mitochondrial (mt) genome gene order in eukaryotes is remarkably high. Depending on evolutionary pathways in different lineages, the types of mt genome organization (linear, circular, or assembled into multiple chromosomes), gene content (involving gene loss and acquisition), and gene arrangement are variable (Sterling-Montealegre, Prada, 2024). The broad range in mt genome sizes (from 11–50 kb in animals to 66 kb–11.3 Mb in plants) is observed largely due to variations in gene number, as well as the length of non-coding mtDNA. The diversity of gene orders (GOs) is determined by both changes in the relative positions of genes and the presence of deletions and duplications. In some major taxa, the GO is conserved (e. g., in vertebrates), while in others it varies widely (e. g., in some groups of invertebrates) (Zardoya, 2020). The gene orders in the mt genomes of crustaceans are among the most diverse found in invertebrates (subphylum Crustacea), which manifested through changes in gene positions relative to the basal order, which is also characteristic of insects (the Pancrustacean pattern, PanGO) (Boore, 1999; Kilpert, Podsiadlowski, 2006; Sterling-Montealegre, Prada, 2024).

The types of gene order alterations can be different. A recent study R.A. Sterling-Montealegre and C.F. Prada (2024) analyzed 299 genomes from the subphylum Crustacea among 464 mt genomes from 47 arthropod orders and identified 87 distinct gene orders, including four gene orders most frequently found in crustaceans.

According to this data, rearrangements in mt genomes of Crustacea that involve tRNA genes accounted for 70.1 % of all structural changes, while alterations in the positions of other genes comprised 29.9 %. Changes in the positions of protein-coding and rRNA genes are less common (Castellucci et al., 2022) than those of tRNA genes, the positions of which can differ even among species within the same family (Jühling et al., 2012). Previous research has demonstrated a positive association between a high rate of gene rearrangement and an elevated nucleotide substitution rate in protein-coding genes of the subclass Caenogastropoda Cox, 1959 (gastropod mollusks) (Fourdrilis et al., 2018). Baikalian amphipods, a group characterized by diverse mt gene order, exhibit significantly accelerated evolution in several protein-coding genes, compared to related freshwater species of the genus Gammarus (Romanova, Sherbakov, 2019). Nevertheless, this correlation is not observed in all groups of organisms (Shao et al., 2003; Xu et al., 2006). Remarkably, the order of genes in mt genomes is highly conserved in some crustacean lineages over long evolutionary timescales, while being highly variable in other groups (Kilpert et al., 2012; Tan et al., 2019; Zardoya, 2020).

Phylogenetic and statistical analyses allow to reconstruct the history of GO changes in different taxa. In some instances, GO can represent informative synapomorphies for particular groups, providing additional support for resolving phylogenetic relationships at the level of infraorders, superfamilies, and families (Tan et al., 2019).

Mt genomes with rearranged gene orders are frequently found in Baikal amphipods (Romanova et al., 2016, 2020; Drozdova et al., 2024). This group of invertebrates originated in the lake through adaptive radiation and has persisted over a long period (Sherbakov, 1999; Mats et al., 2011). The causes leading to GO rearrangements in this group of endemic invertebrates, as well as their putative mechanisms, remain unknown (Mueller, Boore, 2005).

The article investigates the diversity of mt gene order in amphipods through a comparative analysis of gene rearrangements, as well as suggestions on the mechanisms for their origin within an evolutionary framework. The results supported PanGO as the ancestral state for Baikalian species and revealed certain patterns in the emergence of rearrangements within this group. Clades exhibiting divergent GO patterns were also identified among the studied species.

## Materials and methods

Amphipod gene order dataset. Mt genome sequences for this study were retrieved from the GenBank database (Supplementary Material 1)1. The mt genomes of Baikalian species were published in earlier studies (Rivarola-Duarte et al., 2014; Romanova et al., 2016, 2021; Mamos et al., 2021). Specimen collection localities, along with detailed protocols for DNA extraction, PCR amplification, sequencing, genome assembly, and annotation are described in previous publications (Romanova et al., 2016, 2021) and in Supplementary Material 2.

Supplementary Materials are available in the online version of the paper: https://vavilov.elpub.ru/jour/manager/files/Suppl_Sirot_Engl_30_1.zip


The dataset for GO comparison comprised only mt genomes from 127 species which had complete coding sequences of 13 protein-coding genes. Ribosomal and tRNA genes, as well as the control region (CR), were excluded from the dataset because in some species they were either missing or present as duplicated copies, which would have compromised comparative analysis. The mt genome of the Baikalian species Linevichella vortex (Dybowsky, 1874) was also excluded from the GO analysis due to its incomplete genome but was retained for the phylogenetic analysis. For pairwise comparisons, we employed the PanGO scheme. It is important to note that the method of defining gene orders based only on the 13 proteincoding genes (as opposed to all 37 mt genes) more often results in orders being classified as identical, whether they are compared to PanGO or to one another. In contrast, their complete gene orders would not be identical due to the differences in the positions of the remaining genes

Analysis of gene order using the CREx program. The types of mitochondrial gene rearrangements in amphipods were assessed using the program CREx (Common Interval Rearrangement Explorer), which is part of the CREx2 suite (Bernt et al., 2007; Hartmann et al., 2019), on the Galaxy server (The Galaxy Community, 2024) (https://usegalaxy.eu/). The CREx algorithm employs the concept of common intervals – groups of genes that are consecutively arranged in the compared GOs. The number of common intervals (NSCI) is used to assess the similarity of GOs. A higher NSCI value indicates a greater degree of identity between them. Using event models, CREx identified possible rearrangement scenarios, including tandem duplication of a segment of adjacent genes followed by random loss of some gene copies (TDRL), transposition (T), defined as the movement of a gene within the same coding strand of the mtDNA, gene inversion (I) to the other coding strand of the mtDNA, and inverse transposition (iT) (Bernt et al., 2007; Basso et al., 2017).

Within the CREx algorithm, the aforementioned types of rearrangements are assessed exclusively using mathematical models. TDRL is more frequently postulated in scenarios involving larger-scale mt genome rearrangements and the movement of a greater number of genes in a single step compared to transposition. It is also an asymmetric rearrangement that can only occur in one direction for any two compared gene orders, thereby enabling the determination of the ancestral state (Bernt et al., 2007). When two or more alternative scenarios were predicted, the one requiring the smallest number of steps was selected.

Manual counting of position changes in specific genes. In pairwise comparisons of PanGO with the GO of each species, the gene order within gene blocks on both mtDNA strands was examined, along with whether the order of blocks was conserved in the genome. We performed a visual analysis, inspecting the sequence from cox1 to nad1 to identify the boundaries of each conserved block, between which gene rearrangements occurred. Genes occupying the same position as in PanGO were designated as “0”, while individual genes, the position of which was altered relative to the conserved gene blocks in PanGO, were designated as “1” (Supplementary Materials 3 and 4). The quantification results were visualized as histograms, and the corresponding GO schemes were aligned with phylogenetic trees

Phylogenetic reconstructions. For phylogenetic tree construction, amino acid sequences of 13 protein-coding genes from the mt genomes of 128 amphipod species (available in the GenBank database as of December 10, 2023), including 14 Baikalian species (among them the species L. vortex with an incomplete mt genome), and three species from the order Isopoda (Ligia oceanica (Linnaeus, 1767), Eophreatoicus karrkkanj Wilson & Humphrey, 2020, Neomysis japonica Nakazawa, 1910), used as an outgroup, were employed

Amino acid sequences of each protein-coding gene were aligned at the codon level using TranslatorX (Abascal et al., 2010) with the ClustalW algorithm and were subsequently concatenated in SeaView 4.5.4 (Gouy et al., 2010). The optimal amino acid substitution model, JTT+F+I+R9, was selected using ModelFinder (Kalyaanamoorthy et al., 2017). A maximum likelihood phylogenetic tree was constructed with IQ-TREE 2 (Minh et al., 2020) and was subsequently visualized in FigTree 1.4.3 (Rambaut, 2010).

The phylogenetic tree of amphipods, integrated with gene order data, was used to identify regularities of gene rearrangements that emerged during the evolution of the studied taxa. Branches and nodes of the tree where changes in the positions of individual genes occurred, as well as the variants of GOs in different species, were annotated. The correspondence between changes in gene order and species phylogeny was also assessed.

## Results

**Analysis of gene order rearrangements**

**Fig. 1.**
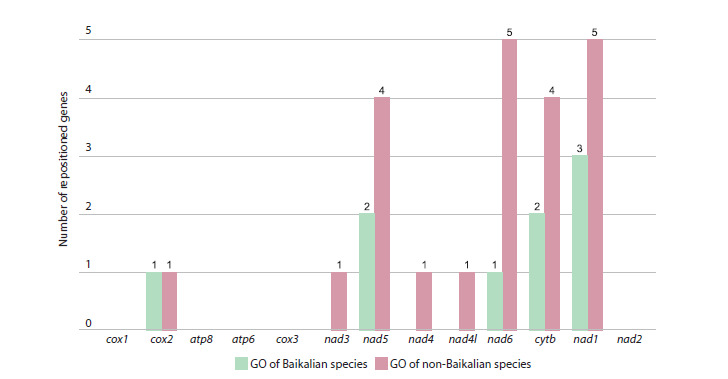
The number of rearrangement events for each mitochondrial protein-coding gene relative to the PanGO, quantified for Baikalian and non-Baikalian amphipod groups.

**Fig. 2.**
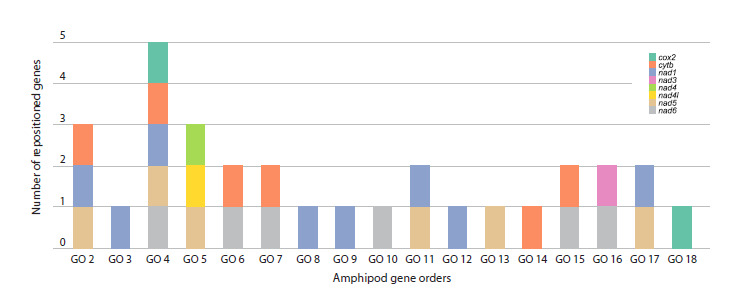
Gene orders of amphipods (GO 2–GO 18) with the number of mitochondrial protein-coding genes repositioned relative to PanGO. GO1, which corresponds to PanGO, is not shown.

Among the 127 complete mt genomes of amphipods analyzed, 18 distinct protein-coding gene orders (GO 1–GO 18) were found. Using CREx, it was determined that a minimum of 1 to 3 rearrangement events are required to transition between different gene orders. Amphipod species, their mt gene orders, and the types of rearrangements are presented in Supplementary Material 3. Further analysis of gene rearrangements was performed considering only unique gene orders. The total number of mt genes that had changed position relative to PanGO was manually quantified and annotated for two groups of amphipods: Baikalian and non-Baikalian species (Fig. 1), as well as separately for each GO (Fig. 2, Supplementary Material 2). Such rearrangements were found to be more frequent in the larger and ecologically more diverse sample of non-Baikalian species.

The nad1 gene was found to be the most frequently repositioned relative to the PanGO (occurring in eight GOs) in both Baikalian and non-Baikalian groups (Fig. 1, Supplementary Material 4). Eight of the thirteen protein-coding genes (nad1, cytb, nad6, nad5, cox2, nad3, nad4, nad4l) underwent rearrangements, particularly inversions, more frequently than others (Fig. 1). The GOs with the highest number of repositioned genes across all amphipods were found in Macrohectopus branickii (Dybowsky, 1874) (GO 4, five genes), in two species of the genus Caprella Lamarck, 1801 and in Cyamus boopis Lütken, 1870 (GO 5, three genes), and Crypturopus tuberculatus with C. inflatus (Dybowsky, 1874) (GO 2, three genes) (Fig. 2, Supplementary Material 3).

Complex rearrangements that involve 2 to 5 repositioned genes were identified in 20 species, corresponding to nine gene orders (GO 2, GO 4, GO 5, GO 6, GO 7, GO 11, GO 15, GO 16, GO 17) (Supplementary Material 4). The remaining GOs had only a single repositioned gene. Gene inversions (I) and inverse transpositions (iT) were frequently observed, occurring in seven of the 17 altered GOs (Supplementary Material 3). Transposition was the most prevalent type of gene rearrangement in amphipod mt genomes, found in 11 GOs.

We classified rearrangement events as complex if they met one of the following criteria: required two or more steps according to CREx calculations; involved a combination of different rearrangement types (inversions, transpositions); or included at least one TDRL event (Bernt et al., 2007; Castellucci et al., 2022). The complex TDRL rearrangement type was found exclusively in Baikalian species M. branickii, C. tuberculatus, and C. inflatus. Non-Baikalian species exhibited combinations of different rearrangement types, but no TDRL events were detected. Only the Baikalian species from lineage I (M. branickii) showed both a high number of rearrangement steps and high event complexity, involving inversion, TDRL, and transposition events. In contrast, the mt genomes of species from lineage II maintain the ancestral PanGO for protein-coding gene order (Supplementary Material 3).

**Phylogenetic analysis**

**Fig. 3.**
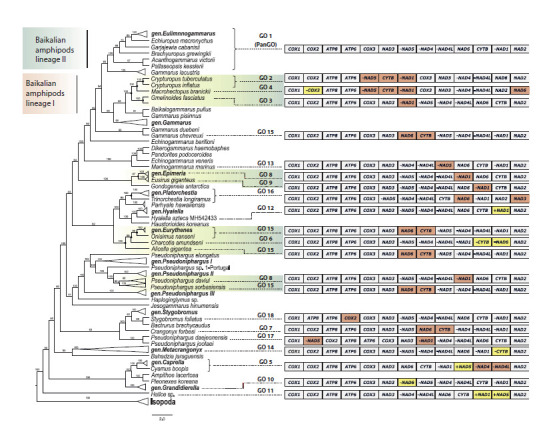
Phylogenetic tree reconstructed from amino acid sequences of 13 mt protein-coding genes from 128 amphipod species, with a scheme of corresponding mt genome gene orders. The tree was manually adjusted for proportions, and branch lengths are scaled. Green-shaded areas indicate species groups with dissimilar GOs. The L. vortex species is not labeled as it was excluded from the GO analysis. Genes that changed their coding strand (I, iT) are highlighted in yellow, while genes subjected to T and TDRL rearrangements are marked in red.

Phylogenetic reconstruction was performed using amino acid sequences of 13 protein-coding genes from 128 amphipod species. The outgroup is represented by a clade of isopods (Fig. 3). The group of Baikalian amphipods includes 13 species. The Baikalian species split into two clades, corresponding to the previously identified lineages I and II.

Each Baikalian lineage clusters with different species from the genus Gammarus Fabricius, 1775: lineage I clusters with G. pisinnus Hou, Li & Li, 2014, G. fossarum Koch, 1836, Baikalogammarus pullus (Dybowsky, 1874) and G. roeselii Gervais, 1835, while lineage II clusters with Gammarus lacustris G.O. Sars, 1863. Therefore, the genus Gammarus is inferred to be paraphyletic. The clustering pattern of the phylogenetic tree indicates that both Baikalian lineages share a common ancestor with species of the genus Gammarus, as G. duebeni Lilljeborg, 1852 and G. chevreuxi Sexton, 1913 occupy a basal position relative to these lineages. Lineage I comprises species from the families Micruropodidae Kamaltynov, 1999, Macrohectopodidae Sowinsky, 1915 and Crypturopodidae Kamaltynov, 2002, while lineage II consists of species from the families Eulimnogammaridae Kamaltynov, 1999, Acanthogammaridae Garjajeff, 1901, and Pallaseidae Tachteew, 2001

Species of the genera Metacrangonyx Chevreux, 1909 (inhabiting marine brackish waters, rarely freshwaters) and Pseudoniphargus Chevreux, 1901 (stygobionts, inhabiting environments from brackish wells to mountain rivers) form monophyletic groups. The species Echinogammarus berilloni (Catta, 1878), found in rivers and streams of Western Europe, occupies a basal position to the clade comprising the Baikalian species and species of the genus Gammarus. The mt gene order of E. berilloni is identical to the GO of the latter species (except for G. chevreuxi).

Integration of phylogenetic and manual gene rearrangement analyses data enabled the identification of the amphipod taxa with the most highly rearranged mt gene orders. Figures 2 and 3 and Supplementary Material 3 demonstrate that the same protein-coding genes have been repositioned in the mt genomes of numerous distantly related species. In some instances, such as in Eurythenes maldoror d’Udekem d’Acoz & Havermans, 2015 and G. chevreuxi (both possessing GO 15), these rearrangements have convergently resulted in identical GOs, representing a case of homoplasy. Analysis of mt genomes from species of the genus Gammarus allowed us to conclude that the PanGO represents a symplesiomorphy for both Baikalian lineages

## Discussion

The results of the gene order analysis in amphipod mt genomes characterize this group as one with frequent and diverse gene rearrangements (18 distinct protein-coding GOs identified across 127 species). Four out of 13 Baikalian species correspond to three distinct GOs, while 53 out of 114 non- Baikalian species correspond to fourteen distinct GOs (Fig. 1 and 2). Thus, GO changes are observed in 57 species within the dataset, which represent 17 distinct GOs. The previously described PanGO is the most common protein-coding gene order among amphipods, although whether it represents the ancestral state for this group remains unconfirmed. The GO 11 found in the basal amphipod species Halice sp. (Li et al., 2019) is significantly different from PanGO.

Amphipods, being an evolutionarily ancient invertebrate group, have presumably accumulated numerous gene order variants (Hou et al., 2014; Mamos et al., 2021). In a study of mt genome diversity across Decapoda infraorders, M.H. Tan et al. determined rearrangement “hotspots” on phylogenetic trees annotated with GO schemes (Tan et al., 2019). These hotspots denote clades of closely related species with two or more unique gene orders that are distinct from one another and from the ancestral state. In total, four groups of amphipods containing dissimilar GOs were identified. Two gene orders (GO 6 and GO 15) were found in the superfamily Lysianassoidea Dana, 1849, with GO 15 involving identical gene rearrangements in six species. Two gene orders (GO 8 and GO 9) were found in the genus Epimeria A. Costa in Hope, 1851, and two (GO 8 and GO 15) in the genus Pseudoniphargus. Three gene orders (GO 2, GO 3, and GO 4) were observed in Baikalian lineage I, with repositioned genes present in four out of its five species (Fig. 3).

**Fig. 4.**
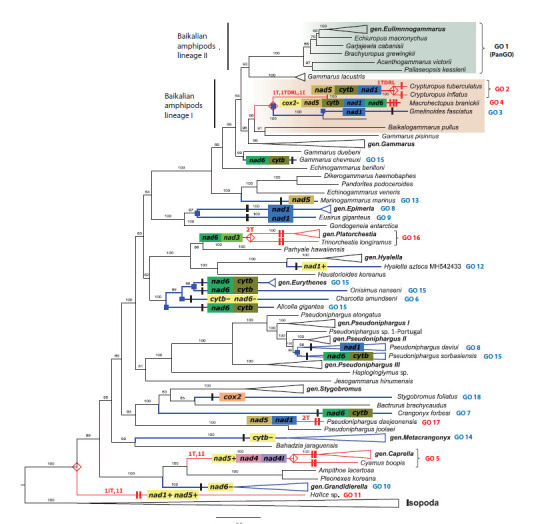
Phylogenetic tree reconstructed from amino acid sequences of 13 mt protein-coding genes from 128 amphipod species. Branches are annotated with the names of genes that have changed position relative to PanGO. Vertical bars on branches show the number of rearrangement steps (CREx). Genes that changed their coding strand (I, iT) are highlighted in yellow. Branches and gene orders marked in red indicate complex rearrangement types within the respective species groups, and those in blue denote simple types of changes. Species L. vortex is not labeled as it was excluded from the GO analysis

Based on CREx calculations, Baikalian lineage I (1–3 steps) shows similarity both to the species group comprising the genus Caprella and the species C. boopis (two steps), and to the basal species Halice sp. (two steps). Gene orders involving more complex rearrangement patterns are concentrated in these groups (Fig. 4). While the formation of GOs in species of the genus Platorchestia Bousfield, 1982 (2T) and the species P. daejeonensis (2T) suggests relatively simpler rearrangement scenarios, which still require two steps. Consequently, Baikalian lineage I represents the group with the highest evolutionary dynamics, demonstrating both the greatest number of complex gene rearrangements and the highest diversity of unique GOs.

Amphipod species with altered GOs, which possess diverse biological and ecological traits, are widely distributed across various taxonomic groups in the phylogenetic tree (Fig. 4), demonstrating the instability of GO as a taxonomic character. Novel gene orders, as well as the ancestral PanGO, are present in both ancient lineages and more recent evolutionary radiations. As expected, there are frequent instances where species from the same family forming a single clade share identical GOs – for example, Caprella mutica Schurin, 1935 and C. boopis (GO 5).

There are also phylogenetically distant amphipod species that show identical GOs when analyzing the protein-coding gene dataset. In particular, homoplasy is observed in three distantly related species groups that share the same rearranged positions of nad6 and cytb genes, corresponding to GO 15 (Supplementary Material 3, Fig. 3 and 4). When tRNA, rRNA, and control regions are included in the analysis, the gene orders of such species are generally no longer identical.

Homoplasies in mt genome gene order are often observed across diverse invertebrate species (Kilpert et al., 2012; Tan et al., 2019; Castellucci et al., 2022). We observed this phenomenon noting both the lower occurrence frequency of proteincoding gene rearrangements compared to tRNA repositioning (Jühling et al., 2012) and its potential role as a convergent trait in distantly related amphipod groups. Similar rearrangement patterns in the same gene cluster nad5-nad4---nad6---nad1 are observed in many freshwater and marine amphipod species. Rearrangements in four Baikalian species also affect this particular gene cluster.

GO in the mt genomes of invertebrates does not always align with their classification into species, genera or other taxa established through morphological characteristics. However, by combining phylogenetic analysis with gene orders comparison, it is possible to identify clades with the highest GO diversity and nodes on the tree where this feature underwent the most substantial modifications (groups with dissimilar GOs). For example, such discordance is observed within species of the genus Pseudoniphargus and among representatives of Baikalian lineage I, where several GOs deviate from the pattern typical for these groups.

Studies report that the uneven distribution of gene rearrangements across the phylogenetic tree is characteristic of Hexapoda and crustaceans, which may be explained by parallel evolution of this trait (Tan et al., 2017; Moreno-Carmona et al., 2021). A representative example includes gene rearrangements in certain cladoceran taxa (e. g., Daphnia, Bosmina) where PanGO is retained in the mt genomes of most but not all the species (17 out of 32 species conform to PanGO, while 15 species exhibit nine other GOs) (Castellucci et al., 2022). While most Gammarus species conform to PanGO, there is a single species, G. chevreuxi, which shows altered cytb and nad6 positions. Even greater gene order divergence is observed in the Hyalella azteca (Saussure, 1858) mt genome with accession MH542433, which contains a nad1 inversion absent in genome MT672041. A previous study reports additional inversions and inverse transpositions within the genus Hyalella S.I. Smith, 1874, which may be associated with distinct southern and northern populations (Zapelloni et al., 2021).Since PanGO is ancestral for both Baikalian lineages, all alterations in GO likely emerged in various Baikalian amphipod species during adaptive radiation within the lake. Mt genome rearrangements appear to have occurred in multiple steps toward the more diverse GOs of lineage I Baikalian species. The least altered gene orders (GO 3, GO 8–10, GO 12–14) represent plesiomorphic states. Lineage I Baikalian amphipods from the families Crypturopodidae, Micruropodidae, and Macrohectopodidae (Kamaltynov, 1999) have highly altered GOs not found in other amphipods. It is also noteworthy that the partial mt genome of L. vortex (Micruropodidae) retains the ancestral PanGO. The species within this group (excluding M. branickii) are shallow-water, thermotolerant amphipods (Gmelinoides fasciatus (Stebbing, 1899), C. tuberculatus, L. vortex) (Kamaltynov, 2001).

Weakened stabilizing selection, resulting from a low effective size and reduced genetic variation in ancestral populations (Charlesworth, 2009; Lavrov, Pett, 2016), may be the factor explaining the increased frequency of GO rearrangements in certain species (Shao et al., 2003). Shallow-water Baikalian amphipods appear particularly prone to rapid population decline due to environmental changes. Specifically, glacial periods during the geological history of Lake Baikal led to cycles of species extinction (Goldberg et al., 2010; Mats et al., 2011), as demonstrated for the southwestern population of G. fasciatus (Bukin et al., 2018) and for M. branickii (Petunina et al., 2023). Furthermore, high microsporidian infection rates observed in G. fasciatus and M. branickii (Petunina et al., 2023) may lead to a decreased proportion of males in the population. Since parasites lack mitochondria and utilize host ATP, it is plausible that adaptation to their negative impact has contributed to mtDNA rearrangements in several amphipod species, which may have improved ATP production efficiency (Bukin et al., 2018).

Amphipod species with completely sequenced mt genomes currently represent diverse taxonomic groups inhabiting ecologically distinct biotopes in the lake. A number of authors have attempted to define whether associations exist between GO variations and ecological characteristics, divergence times, features of life cycle or habitat, concluding that GO changes are multifactorial and may be linked to evolution in a number of biochemical and metabolic traits (Romanova, Sherbakov, 2019; Tan et al., 2019; Castellucci et al., 2022; Benito et al., 2024). In a study of the isopod species Janira maculosa Leach, 1814, the authors suggested that the frequently observed gene rearrangements in the mt genome could be partially explained by its low structural complexity compared to the nuclear genome (Kilpert et al., 2012). Gene relocations, particularly to the opposite coding strand, affect the mitochondrial transcription process and may influence its efficiency.

Lineage II of Baikalian amphipods is a group with greater taxonomic and ecological diversity that maintains the ancestral protein-coding GO. Nevertheless, interspecific differences exist in the number and organization of tRNA genes (Romanova et al., 2016; Romanova, Sherbakov, 2019). It has been proposed in some studies that changes in tRNA gene number and position occur more often than ones affecting rRNA or protein-coding genes in mt genomes (Pääbo et al., 1991; Jühling et al., 2012).

One hypothesized mechanism for mt gene changes is partial or complete genome duplication, followed by subsequent loss of duplicated regions (Jühling et al., 2012). Existing models for calculating scenarios of converting one GO into another are based on the assumption that an equal number of duplication and deletion events occurs at each step of the TDRL process. Although the distance calculation between two gene orders under the TDRL model is known to be asymmetric, requiring a different number of steps for forward and reverse scenario reconstructions (Bernt et al., 2007), the model does not account for the possibility that the ratio of transformations during TDRL (e. g., one duplication to several deletions, or vice versa) could be more complex. Consequently, the inferred minimal number of steps for a rearrangement may overestimate those actually required by the true evolutionary scenario.

The replication of mtDNA via the rolling circle mechanism, which produces a dimeric mtDNA molecule (Fučíková et al., 2016; Xia et al., 2016; Wang et al., 2022), along with deletion events affecting either copy, may result in the duplication of tRNA genes and protein-coding gene clusters. Various mechanisms for the formation of such deletions involving either recombination or errors in replication have been described in diverse taxonomic groups (Nissanka et al., 2019; Oliveira et al., 2020). Furthermore, if a promoter mutation occurs within one copy of mtDNA dimer, it may lead to its pseudogenization (Lavrov et al., 2002).

Indirect evidence for the presence of such a mechanism in Baikalian species includes a duplicated cox2 gene and three fragmented atp8 copies in M. branickii, along with truncated gene copies within extensive non-coding regions of G. fasciatus (atp6, nad4l), Brachyuropus grewingkii (Dybowsky, 1874) (atp8, cox2, nad2), Garjajewia cabanisii (Dybowsky, 1874) (cytb), including duplicated tRNA genes (Romanova et al., 2020). These regions, typically uncommon in animal mt genomes, may indicate historical duplication events followed by subsequent sequence degeneration (Boore, 1999), while potentially serving as a factor facilitating further gene rearrangements.

In certain cases, mt gene order may provide supplementary diagnostic characters for taxonomic classification (Lavrov, Lang, 2005; Tan et al., 2019). However, explaining the underlying causes of accelerated mtDNA evolutionary rate and the high variability of GOs observed in both Baikalian and other amphipod species will require further investigation.

## Conclusion

Analysis of protein-coding gene order in the mitochondrial genomes of Baikalian amphipods allowed us to confirm PanGO as the ancestral state for both Baikalian lineages. Gene order alterations in several Baikalian species emerged during their evolutionary history within the lake. The rearrangement pathways inferred by CREx likely represent simplified models, as the underlying duplication and deletion events may occur with unequal probabilities.

Analysis of gene order rearrangements and phylogenetic analysis of protein-coding sequences of available amphipod mt genomes revealed four distinct groups with complex gene rearrangement patterns. These comprise clades that include species from the superfamily Lysianassoidea, the genus Epimeria, the genus Pseudoniphargus, and Baikalian amphipods of lineage I. We propose that the more ancient origin of lineage I Baikalian species relative to lineage II may partially explain their greater diversity in gene order arrangements. Additionally, it is hypothesized that the low effective population size in lineage I Baikalian amphipods could be one of the factors that is weakening the effect of stabilizing selection, thereby enabling the fixation of mt genomic rearrangements.

## Conflict of interest

The authors declare no conflict of interest.
